# Long-Term Outcomes of Peripheral Blood Mononuclear Cells in the Treatment of Angiitis-Induced No-Option Critical Limb-Threatening Ischemia

**DOI:** 10.3389/fcvm.2021.769472

**Published:** 2021-12-06

**Authors:** Xiaolang Jiang, Hao Liu, Tianyue Pan, Shiyang Gu, Yuan Fang, Zheng Wei, Gang Fang, Bin Chen, Junhao Jiang, Yun Shi, Peng Liu, Weiguo Fu, Zhihui Dong

**Affiliations:** ^1^Department of Vascular Surgery, Institute of Vascular Surgery, Zhongshan Hospital, Fudan University, Shanghai, China; ^2^Department of Hematology, Zhongshan Hospital, Fudan University, Shanghai, China

**Keywords:** adult stem cells, angiogenesis, autologous stem cell transplantation, CD34+, cell transplantation

## Abstract

**Background:** Peripheral blood mononuclear cells (PBMNCs) showed encouraging short outcomes in the treatment of angiitis-induced no-option critical limb-threatening ischemia (AICLTI) in the pilot study. This study aimed to demonstrate the long-term outcomes of this treatment.

**Methods:** From May 2014 to December 2018, patients diagnosed with AICLTI and treated by autotransplantation of PBMNCs in our center were enrolled and analyzed. The primary endpoint was major amputation-free survival (MAFS), the secondary endpoints included peak pain-free walking time (PPFWT), Wong-Baker FACES pain rating scale score (WFPRSS), labor recovery, ankle-brachial index (ABI), transcutaneous partial oxygen pressure (TcpO2), and SF-36v2 scores.

**Results:** A total of 58 patients were enrolled. During a minimal follow-up of 36 months, the MAFS was 93.1% and the labor competence restored rate was 62.1%. The WFPRSS was decreased from 8.7 ± 1.6 to 1.6 ± 3.2, and PPFWT was significantly improved from 2.9 ± 4.2 min to 16.6 ± 6.9 min. The quality of life was also significantly improved at each follow-up point. Perfusion evaluating parameters, such as ABI and TcPO2, were also significantly improved. No critical adverse event was observed during the treatment and follow-up period.

**Conclusions:** The treatment of AICLTI by autotransplantation of PBMNCs demonstrated encouraging long-term results. It could not only restore labor competence, improve the quality of life, but also significantly reduce the major amputation rate.

## Introduction

Angiitis-induced critical limb-threatening ischemia (AICLTI) was identified to be different with atherosclerosis obliterans (ASO). Patients with AICLTI were usually younger, involved the middle and distal vessels, and the angiitis was the main pathogeny (1, 2), which made the mainstream vascular reconstruction difficult to be performed or with poor outcomes. Thromboangiitis obliterans (TAO) accounted for more than 90% of the patients with AICLTI, the primary and secondary patencies after bypass procedure were 41 and 54% at 1 year, 32 and 47% at 5 years, and 30 and 39% at 10 years, respectively (3). Studies, such as peripheral blood mononuclear cells (PBMNCs), bone marrow-derived stem cells (BMDSCs), and purified CD34+ cells (PCCs) in the treatment of AICLTI have been reported (4). However, most of the previous studies focused on the patients with ASO or diabetes mellitus and were absent from long-term outcomes (5, 6). Of note is the fact that most of the previous studies merely involved a few to a dozen patients with AICLTI (7–11). In our previous randomized controlled trial (RCT), comparing the PBMNCs with PCCs in the treatment of AICLTI, the 3-year outcomes of PBMNCs also have been reported (12). Compared with BMDSCs and PCCs, the extraction and collection of PBMNCs were simpler and more cost-effective. Despite this, the sample size of this RCT was too small and the long-term outcome of PBMNCs needed to be further elucidated with a larger sample size. We herein conducted this study to report the long-term clinical outcome of AICLTI treated by PBMNCs with more patients and a minimal follow-up of 36 months.

## Materials and Methods

### Patient Enrollment

From May 2014 to July 2018, patients diagnosed with AICLTI and treated by autotransplantation of PBMNCs in our center were enrolled and analyzed. The definition of TAO was according to Olin (2). The study was carried out according to the principles of the Declaration of Helsinki and was approved by the Ethics Committee of the Zhongshan Hospital, Fudan University. Informed consent was obtained from all eligible patients, and their medical records were reviewed. The inclusion and exclusion criteria had been elucidated previously (4). Patients should meet all the following criteria: (1) aged 18–80 years; (2) stenotic or occlusive lesions in the limb arteries confirmed by CT angiography (CTA), magnetic resonance angiography (MRA), or digital subtraction angiography (DSA); (3) Rutherford classification four or five unsuitable for open surgery or endovascular therapy; (4) no improvement for at least 3 months after open surgery or endovascular treatment, or at least 1 month of conservative treatment, such as smoking cessation, drug therapy, and exercise therapy; and (5) an unhealing ulcer at least 1 month after optimal care. Exclusion criteria included (1) serious health events that occurred <3 months before admission, including but not limited to myocardial infarction, cerebral apoplexy, pulmonary embolism, severe hepatic dysfunction, and renal dysfunction; (2) or contraindications for the administration of the granulocyte colony-stimulating factor (G-CSF); and (3) no informed consent signed.

### Mobilization and Transplantation

The detailed treatment procedure had been described previously (4, 13, 14), and it was summarized as follows: the G-CSF was mobilized for 5 days, during which low molecular weight heparin (4100 IU) was injected subcutaneously each day. Meanwhile, the blood test was performed every single day to check the count of white blood cells (WBC) and CD34+ cells. On the fifth day, apheresis (COM.TEC; Fresenius HemoCare, Friedberg, Germany) was performed. The final cell products were assessed by leukocyte counting and flow cytometry using CD34 antibodies. The implanted CD34+ cell doses were ensured ranged from 10^5^ to 10^6^ per kg body weight. Cell transplantation was performed by intramuscular injection under general anesthesia with laryngeal mask anesthesia. A total of 80 and 160 sites were each injected with 0.5 ml. In addition, the injections were distributed in the calf and foot or the forearm and hand of the ischemic limbs evenly. Debridement and minor amputation were not routinely performed unless the patient was complicated with wet gangrene or serious infection. Patients with autoimmunological diseases were administered regular immunological treatment and follow-up by rheumatologists. The patients were given aspirin (100 mg/d), cilostazol (400 mg/d), and anplag (300 mg/d) for at least 1 year after the operation. In addition, analgesic therapy was not usually recommended because most of the pain would alleviate within 1 month.

### Follow-Up and Data Collection

All patients were followed once a month for the first 3 months, and every 3 months for the rest of the first year, and once a year thereafter. The primary endpoint was major amputation-free survival (MAFS), which was defined as amputation above the joint. The secondary endpoints included (1) peak pain-free walking time (PPFWT), which was measured on a treadmill at 2.5 km/h and 10% incline at 0, 3, 6, 12, and 36 months, (2) Wong-Baker FACES pain rating scale score (WFPRSS), which was used to test the pain of the patients at 0, 1, 3, 6, 12, and 36 months, (3) labor recovery, which was defined as returning to original work of patients who were unemployed or suspended, (4) ankle-brachial index (ABI) and transcutaneous partial oxygen pressure (TcPO2) of the treated limb were recorded at 1, 3, 6, 12, and 36 months, and 36-item Short-Form Health Survey version 2 (SF-36v2) score (15), which was used the evaluated the quality of life (QoL) at 0, 12, 24, and 36 months. Ulcer healing is confirmed by a clinician who observed and photographed the limbs of the patients. The definitions of recurrence and ischemia onset were detailed in the previous article (13).

### Statistical Analysis

Data were collected using Microsoft Excel (Microsoft, Redmond, WA, USA) and analyzed by GraphPad Prism (GraphPad Software Inc., La Jolla, CA, USA). Continuous variables were presented as mean ± SD, and categorical variables were presented as frequencies and percentages. The Student's *t*-test or Mann-Whitney U-test was applied to test the differences at different follow-up points for continuous variables depending on the distribution of the data. MAFS was calculated by Kaplan-Meier analysis.

All *P-*values were two-tailed, and a *P* < 0.05 was defined as statistically significant.

## Results

From May 2014 to July 2018, a total of 58 patients underwent transplantation of PBMNCs in our center. The mean age was 41.9 ± 13.0 years with males accounting for 100%. The etiology was TAO in 56 (96.6%), systemic lupus erythematosus (SLE) in 1 (1.7%), and eosinophilia in 1 (1.7%) patient. The mean follow-up time was 56.4 ± 16.0 months, and the median numbers of CD34+ cells were 8.5 × 10^5^ cells/kg [the interquartile range (IQR): 5.1–11.7 × 10^5^ cells/kg] (Table 1).

**Table 1:** Baseline characteristics of patients.

	**X ± SD or No. (%)**
Age	41.9 ± 13.0
Male	58 (100)
Etiology	
Thromboangiitis obliterans	56 (96.6)
Eosinophilia	1 (1.7)
Systemic lupus erythematosus	1 (1.7)
Rutherford classification	
4	3 (5.2)
5	55 (94.8)
Comorbidities	
Smoking	58 (100)
Hypertension	2 (3.4)
Hyperlipidemia	2 (3.4)
Coronary artery disease	1 (1.7)
Diabetes mellitus	
Medication history	
Aspirin	50 (86.2)
Beraprost sodium	45 (77.6)
Cilostazol	20 (34.5)
Clopidogrel	6 (10.3)
Warfarin	2 (3.4)
Rivaroxaban	2 (3.4)
Treatment history	
Catheter-directed thrombolysis	20 (34.5)
Percutaneous balloon angioplasty	18 (31.0)
Stenting	5 (8.6)
Embolectomy	3 (5.2)
Bypass	3 (5.2)
Lumbar sympathectomy	2 (3.4)

*SD, standard deviation*.

### All-Cause Mortality and Amputation

**Figure 1:**
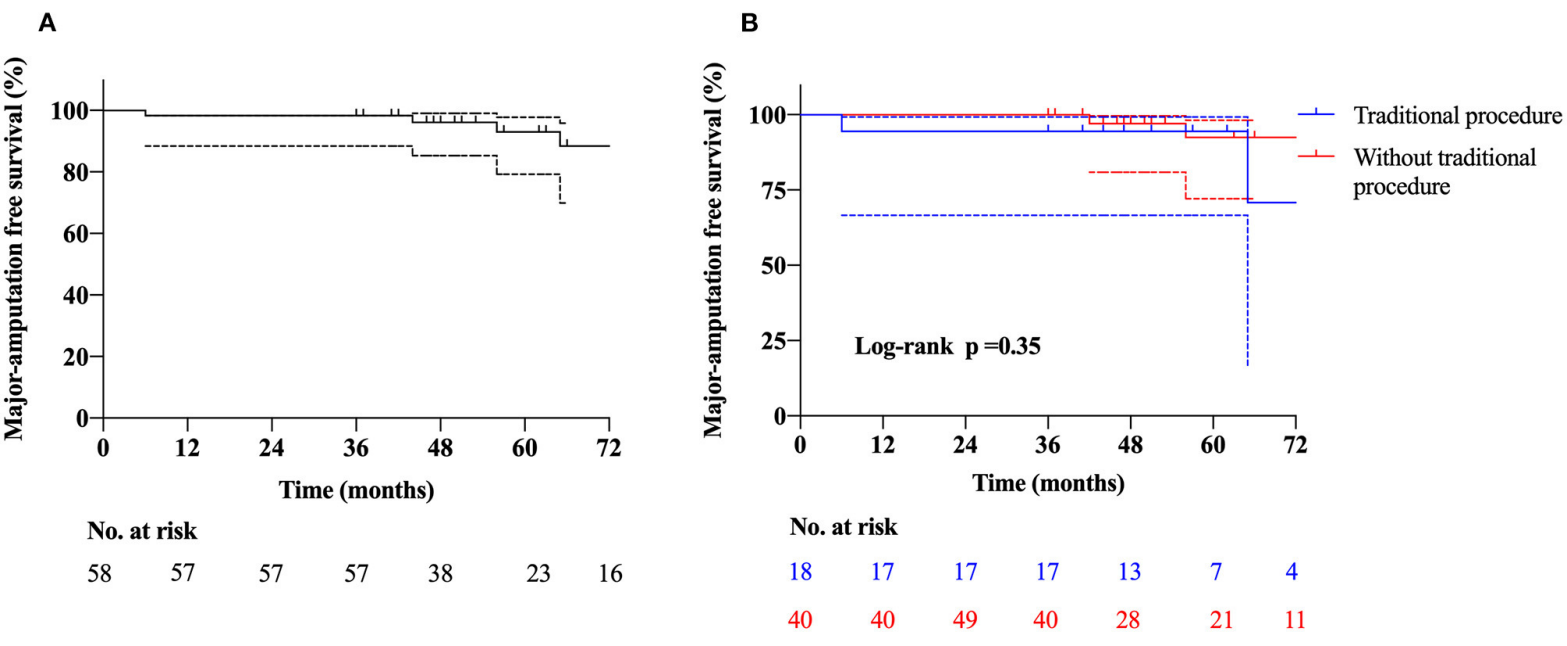
Kaplan-Meier curves show the rates of **(A)** major amputation free survival, **(B)** major amputation free survival in patients with and without traditional procedures.

One patient died 18 months after transplantation, which was identified to be irrelevant to the intervention. Major amputation occurred in four (6.9%) patients. Patient one was a 44-year-old male with a more than 10-year history of CLTI in the right lower extremity. He underwent lumbar sympathectomy and bypass procedure before treatment of PBMNCs; however, the symptom was not alleviated. Although the pain was significantly relieved after transplantation, the ulcer in the foot was unhealed. He underwent amputation above the knee 6 months later. Patients two and three were all Rutherford classification 5, and they underwent amputation above the knee at 3 and 2 months after transplantation due to unbearable pain, respectively. Patient four was a 50-year-old man who underwent amputation above the knee 3 months later due to an unhealed ulcer. Amputation of toes was performed in five (8.6%) patients. Among them, three patients were performed along with the transplantation, and the other patients underwent the amputation within 1 month. The 36-month MAFS was 93.1% (Figure 1A).

### Ulcer Healing and Pain Alleviation

**Figure 2:**
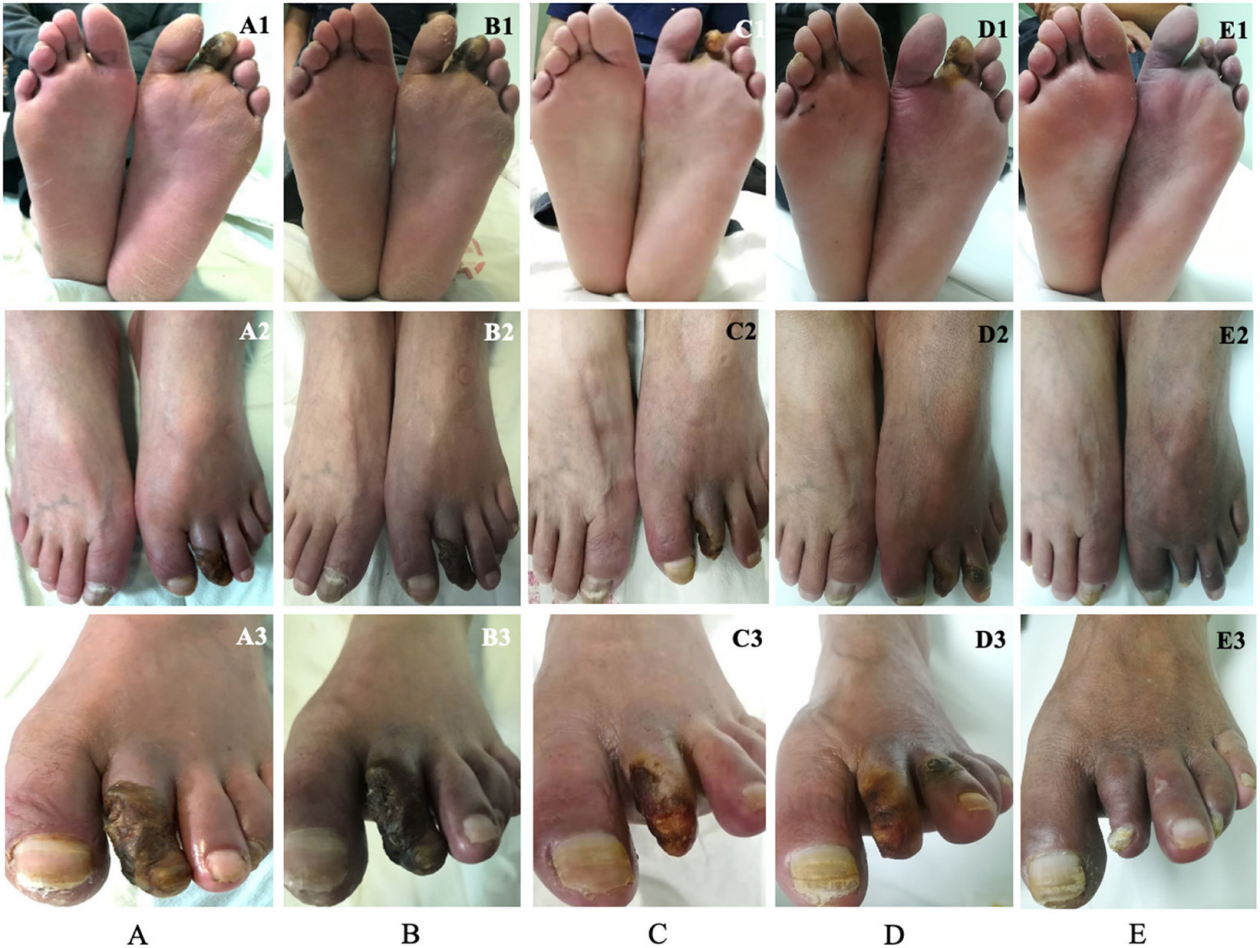
A 55-year-old man was diagnosed with TAO and the progress of ulcer healing during the 12-month follow-up, which was photographed in three different angles. Before transplantation of PBMNCs, this patient presented gangrene in the second toe of the left foot **(A)**. The swelling around the gangrene and the rest pain decreased significantly at 1 month **(B)**. The pain disappeared, the necrotic tissue, and the toenail of the toe dropped at 3 months, only a little ulcer remained **(C)**. At 6 months, the remaining ulcer in the second toe was completely healed, and the patient returned to work. However, the ulcer appeared in the third toe **(D)**. The toenail of the second toe grew in again and the ulcer in the third healed completely **(E)**. TAO, thromboangiitis obliterans; PBMNCs, peripheral blood mononuclear cells.

**Figure 3:**
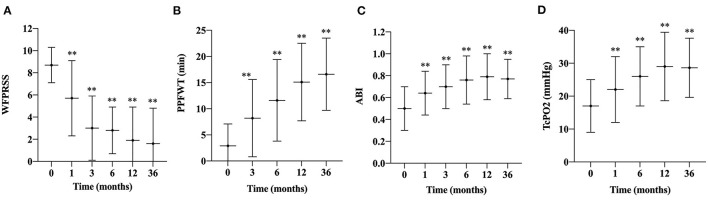
Changes in WFPRSS, PPFWT, ABI, and TcPO_2_ during the follow-up. Changes in the WFPRSS **(A)**, PPFWT **(B)**, ABI **(C)**, TcPO_2_, **(D)** in patients after treatment of PBMNCs are depicted as linear graphs with mean values and the SD bars. (**, *P* < 0.01). WFPRSS, Wong-Baker FACES pain rating scale score; PPFWT, peak pain-free walking time; ABI, ankle-brachial index; TcPO_2_, transcutaneous partial oxygen pressure; SD, standard deviation.

Before transplantation, three and 55 patients were categorized as Rutherford classification four and five, respectively. Fifty-five (94.8%) patients had ulcers on the feet, and 48 (87.3%) of them healed during the follow-up period (Figure 2). The mean time of healing was 6.1 ± 3.7 months. The mean time of pain alleviation was 1.7 ± 1.2 months. The WFPRSS showed significant pain alleviation at each follow-up point and remained persisted until a minimal follow-up of 36 months. The WFPRSS was decreased from 8.7 ± 1.6 at baseline to 1.6 ± 3.2 at 36 months (Figure 3A).

### Efficacy Evaluation

After transplantation, the PPFWT was significantly improved at each follow-up time point compared with baseline measurement (2.9 ± 4.2 min) and remained persisted till 36 months (16.6 ± 6.9 min) (Figure 3B).

**Figure 4:**
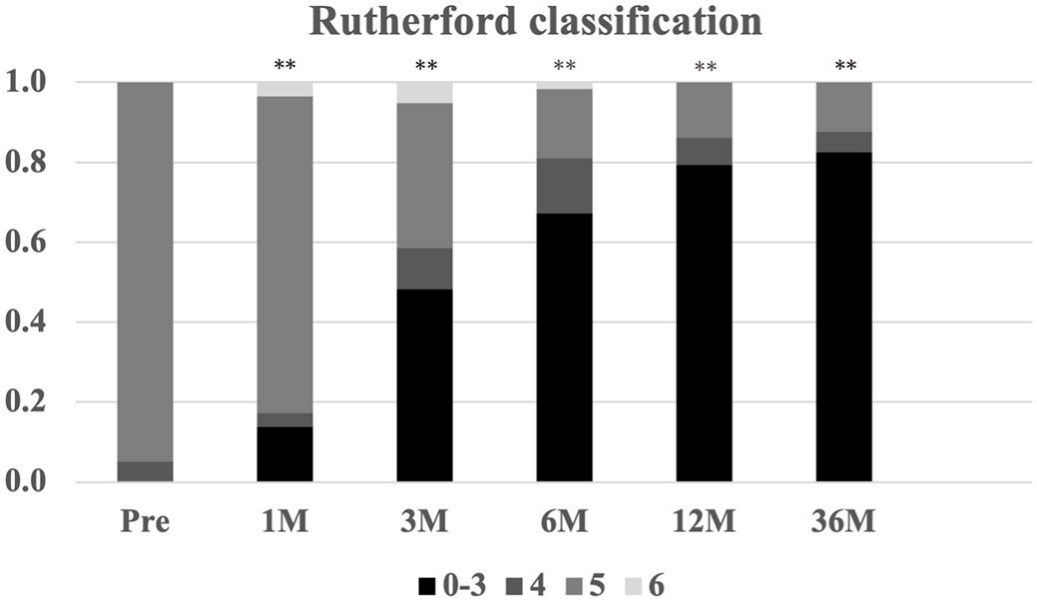
The change in Rutherford classification during the 12-month follow-up. Serial changes in Rutherford classification (0–6) proportions **P < 0.01 vs. baseline.

Regarding perfusion evaluating parameters, mean ABI and TcPO2 were also significantly improved from 0.5 ± 0.2 and 22.0 ± 10.0 mmHg at baseline to 0.8 ± 0.2 and 28.6 ± 9.0 mmHg at 36 months, respectively (Figures 3C,D). In addition, the Rutherford classification was also significantly decreased during the follow-up period (Figure 4).

**Figure 5:**
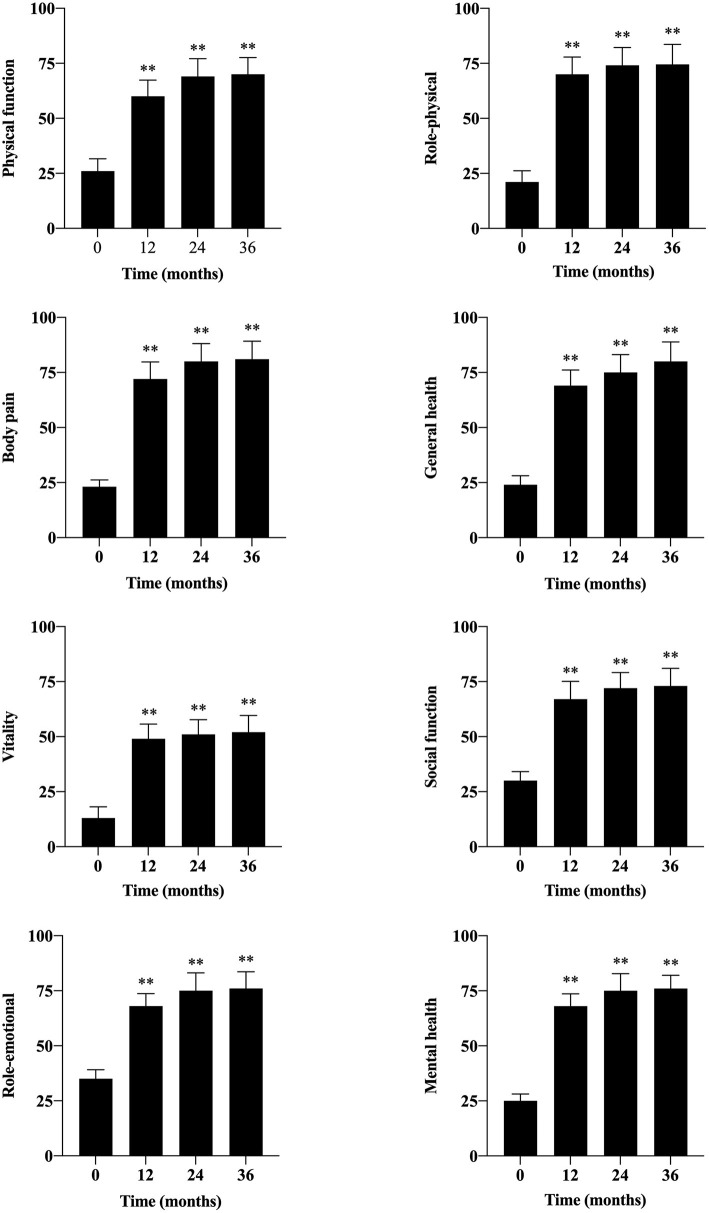
Evaluation of quality of life by SF-36v2 during the follow-up period. The SF-36v2 included eight aspects: physical function, role-physical, body pain, general health, vitality, social function, role-emotional, and mental health. (**, *P* < 0.01).

Quality of life, evaluated by SF-36v2, was significantly improved at each follow-up time (Figure 5). Before PBMNCs transplantation, all of them lost jobs or quitted working temporarily, and 36 of them restored labor competence after transplantation, and the labor competence recovery rate was 62.1% at 36 months.

### Safety Evaluation

No critical adverse events occurred during the treatment and follow-up period. The counts of WBC recovered to a normal level within 1 week in all patients after transplantation. Five patients suffered from adverse events during the mobilization period, such as one patient with a slight fever, two patients with back pain, one patient with transient headache, and one patient with pruritus. All of them completely recovered within 2 weeks after transplantation. Pathological retinal angiogenesis was not observed during the follow-up period.

## Discussion

Angiitis-induced no-option critical limb-threatening ischemia has a wide spectrum of etiologies, such as TAO, SLE, eosinophilia, scleroderma, Crohn's disease, and Sjogren's syndrome. TAO is the most common cause accounting for more than 90% of the patients and associated with the major amputation rate of more than 31% with conventional treatment (3, 16). Meanwhile, AICLTI is characterized by a higher incidence in China than that in the West and a higher proportion among no-option patients. Among the 58 patients enrolled, before transplantation, catheter-directed thrombolysis was performed in 20 patients (34.5%), percutaneous balloon angioplasty in 18 patients (31.0%), stenting in five patients (8.6%), embolectomy in three patients (5.2%), a bypass in three patients (5.2%), and lumbar sympathectomy in two patients (3.4%). However, these mainstream treatments, aiming at ASO-induced CLTI, failed to alleviate ischemia of these patients, which also indicated that the differences between AICLTI and ASO-induced CLTI. After treatment of PBMNCs, the MAFS was 93.1% at 3 years, which was not significantly different in the subgroup without previous conventional procedures (Figure 1B). Meanwhile, the PPFWT increased from 2.9 ± 4.2 min at baseline measurement to 16.6 ± 6.9 min at 36 months, and the WFPRSS was significantly decreased from 8.7 ± 1.6 at baseline to 1.6 ± 3.2 at 36 months. In addition, QoL was significantly improved in all aspects. The results demonstrated the efficacy of PBMNCs in the treatment of AICLTI, even for patients having undergone failed traditional therapy.

Therapeutic angiogenesis was demonstrated to be a kind of innovated and promising method for no-option CLTI, and its efficacy had been confirmed by lots of studies (4, 13, 17–19). However, most of the previous studies focused on the patients with ASO or diabetes mellitus and merely included a large sample size of patients with AICLTI (5–11). The most common used methods were vascular endothelial growth factor (VEGF)-related gene transfer (20, 21), bone-marrow mononuclear cells (BMMNCs) (17, 22–25), PBMNCs (6, 26–29), or PCCs transplantation (13, 19). In the initial cell therapy, 400–500 ml bone marrow would be acquired by bone marrow puncture to get the total number of mononuclear cells in (1–3) × 10^9^, and 100–200 ml bone marrow should be extracted even after G-CSF mobilization. Kawamoto confirmed that the number of mononuclear cells mobilized by G-CSF in peripheral blood was similar to that of bone marrow in 2009 (19). After that, plenty of studies concerning G-CSF mobilized PBMNCs have been reported. Horie et al. performed a randomized, large-scale clinical trial of G-CSF mobilized autologous PBMNC transplantation revealed that cell therapy was feasible and tolerable in patients with both mild and severe peripheral artery disease (PAD); however, this study only included nine patients with TAO followed by 12 months (10). In our previous RCT, we firstly compared the effectiveness between PBMNCs and PCCs. Although the 1- and 3-year outcomes demonstrated that PCCs were not inferior to PBMNCs at limb salvage in the treatment of AICLTI and appeared to induce earlier ischemia relief, PCCs were not more cost-effective than PBMNCs (4, 12). Furthermore, PBMNCs were more suitable for patients with two or more critically ischemic limbs, given more CD34+ cells needed, whereas PCC transplantation would lose a substantial number of CD34+ cells during the isolation. Additionally, a smaller number of CD34+ cells (/body weight) was proved to be associated with reduced overall survival in patients with lower limb ischemia (11). Avoidance of the isolation process also made PBMNCs more simple than PCCs. Meanwhile, the separated CD34– cells were also confirmed to contribute to angiogenesis synergistically via paracrine effects. It was unclear whether removal of CD34– fraction could result in beneficial or adverse influence on the efficacy of transplantation of mononuclear cell (30, 31). In this study, the median numbers of CD34+ cells were 8.5 × 10^5^ cells/kg, which was smaller than patients who underwent transplantation of BMMNCs. We enrolled 58 patients with AICLTI, the 3-year MAFS was 93.1%, and 36 (62.1%) patients restored their labor competence, which was significantly higher than reported traditional procedures (3, 16). Based on these advantages, PBMNC was an easy and effective method to develop to treat AICLTI in clinical practice.

As the most common cause of AICLTI, TAO accounted for more than 90% of the patients (3). Moreover, the 5-year cumulative survival rate was about 97%, which was higher than ASO (16). Besides, patients with AICLTI were younger than patients with ASO and 34.8% of them would lose labor competence before 42 years (16, 32). Hence, it is of great significance and social value to find effective and durable new therapies and to avoid major amputation and loss of labor competence, especially in China where there is a relatively large population of patients with AICLTI (33–35). In the current study, 96.6% (56/58) of patients were diagnosed as TAO, and the mean age was only 41.9 ± 13.0 years. Before treatment, all of them lost jobs. Inspiringly, 36 of them restored labor competence after treatment of PBMNCs. Although the other patients were not competent to work, they could take care of themselves in daily life, which was also a great relief for their families.

Although antiplatelet, anticoagulant, statins, vasodilators, and smoking cessation are widely used in the treatment of patients with TAO, none of these treatments provided noticeable improvement (32, 36). In addition, Fazeli et al. concluded that long-term treatment with angiogenic medication may be a risk factor for dermal gangrene in patients with TAO and ultimately might be a disease management double-edged sword (37). Fang et al. found that smoking cessation does not make a big difference to the symptoms of the patients when they presented CLTI. There was no significant difference between patients with and without smoking cessation in terms of the major amputation rate, the PPFWT, the WFPRSS, the ulcer healing rate, or the recurrence rate after cell therapy (13). All the patients in this study underwent at least 1 month of smoking cessation before transplantation, and no improvement was observed. On the contrary, their ischemic symptoms were significantly alleviated, and ulcer healing was observed in 48 (87.3%) patients. The self-contrast indicated the efficacy of PBMNCs in the treatment of AICLTI. Although repetitive education, quitting smoking was not achieved in all patients. Recurrence and ischemia onset occurred in four (6.9%) and one (1.2%) patients, respectively. All of them were active smokers, indicating smoking may play a critical role in recurrence after transplantation, the difference was not significant probably due to the sample size, however. The mean time from transplantation to recurrence was 14.6 ± 8.5 months. These patients underwent second transplantation and satisfactory outcomes were yielded. Patients with AICLTI definitely benefit from quitting smoking in the long-term follow-up; however, smoking cessation alone is not adequate for the limb salvage in TAO patients with CLTI. The revascularization was mandatory to relieve ischemia, reduce amputation rate, and improve long-term QoL.

Unlike conventional procedure, although the efficacy and validity of PBMNCs in the treatment of AICLTI have been demonstrated by plenty of studies, the potential mechanism still needed to be elucidated. Asahara et al. demonstrated that endothelial-cell progenitors were present as CD34 + hematopoietic stem cells in human peripheral blood and that transplantation of CD34 + instead of CD34– cells promoted angiogenesis in animal models of lower-limb ischemia (31). Miyamoto confirmed that the transplanted endothelial progenitor cells (EPCs) gathered on the outside of the neovessels, meanwhile, the VEGF was also detected (38). The same phenomenon was also confirmed by Kawamoto et al. in an ischemic swine model (39). On the other hand, there were also many studies that reported that EPCs could directly participate in angiogenesis in ischemic limb and myocardial tissues (40, 41). The vasculogenesis induced by EPCs and the angiogenesis stimulated via the paracrine effects of transplanted cells are major contributors to perfusion improvements and limb salvage (42–45). Moreover, the purity of CD34+ cells is directly proportional to their angiogenic efficacy because a higher level of purity promotes angiogenesis. The CD34– cells may promote angiogenesis by paracrine effects, the in?ammatory reaction induced by CD34– cells was also reported, however (45, 46). The question of whether CD34-induced inflammation or promoted angiogenesis remains unclear.

There were several limitations of this study. First, this was a retrospective study and complied with its own nature. Second, although plenty of evaluation index was applied in this study just as in the previous studies (12, 13, 18, 19), there was still a lack of “gold standard” for evaluation of the efficacy of the PBMNCs. Meanwhile, ABI usually reflects the blood flow to the ankle joint, which may not reflect the therapeutic angiogenesis, and the new evaluation method, such as volumetric enhanced CT, maybe more useful. Third, there was no marker indicating the inflammation in these patients, which could forecast the disease progression and clarify the relationship between inflammation and PBMNCs. Despite all these, the current study does provide practical and useful information for the management of AICLTI by PBMNCs.

## Conclusion

This study demonstrated that the long-term outcomes of transplantation of PBMNCs in the treatment of AICLTI were encouraging and durable. It could not only reduce the major amputation rate but also significantly restore labor competence, improve the quality of life. Meanwhile, the technical requirements of this operation are relatively low, which allows easier promotion and development in different hospitals. The conclusion needs to be further confirmed by more cases and longer follow-up.

## Data Availability Statement

The raw data supporting the conclusions of this article will be made available by the authors, without undue reservation.

## Ethics Statement

The studies involving human participants were reviewed and approved by Ethics Committee of Zhongshan Hospital, Fudan University. The patients/participants provided their written informed consent to participate in this study. Written informed consent was obtained from the individual(s) for the publication of any potentially identifiable images or data included in this article.

## Author Contributions

XJ, HL, TP, WF, and ZD: conception and design, writing the manuscript, and overall responsibility. XJ, HL, TP, SG, YF, WF, and ZD: analysis and interpretation. XJ, ZW, GF, BC, JJ, YS, and PL: data collection. ZW, GF, BC, JJ, YS, PL, WF, and ZD: critical revision. XJ, HL, TP, SG, YF, ZW, GF, BC, JJ, YS, PL, WF, and ZD: final approval of the article. XJ, HL, TP, SG, and YF: statistical analysis. WF and ZD: obtained funding. All authors contributed to the article and approved the submitted version.

## Funding

This work was funded by National Nature Science Funds (81970407), the Training Program for Outstanding Academic Leaders of the Shanghai Health and Family Planning System (2018BR40); the Project of Outstanding Academic Leaders of Shanghai Science and Technology Commission (19XD1401200).

## Conflict of Interest

The authors declare that the research was conducted in the absence of any commercial or financial relationships that could be construed as a potential conflict of interest.

## Publisher's Note

All claims expressed in this article are solely those of the authors and do not necessarily represent those of their affiliated organizations, or those of the publisher, the editors and the reviewers. Any product that may be evaluated in this article, or claim that may be made by its manufacturer, is not guaranteed or endorsed by the publisher.
